# Remotely Sensed High-Resolution Global Cloud Dynamics for Predicting Ecosystem and Biodiversity Distributions

**DOI:** 10.1371/journal.pbio.1002415

**Published:** 2016-03-31

**Authors:** Adam M. Wilson, Walter Jetz

**Affiliations:** 1 Department of Geography, University at Buffalo, Wilkeson Quad, Buffalo, New York, United States of America; 2 Department of Ecology and Evolutionary Biology, Yale University, New Haven, Connecticut, United States of America; 3 Department of Life Sciences, Imperial College London, Silwood Park, Ascot, United Kingdom; Centre National de la Recherche Scientifique, FRANCE

## Abstract

Cloud cover can influence numerous important ecological processes, including reproduction, growth, survival, and behavior, yet our assessment of its importance at the appropriate spatial scales has remained remarkably limited. If captured over a large extent yet at sufficiently fine spatial grain, cloud cover dynamics may provide key information for delineating a variety of habitat types and predicting species distributions. Here, we develop new near-global, fine-grain (≈1 km) monthly cloud frequencies from 15 y of twice-daily Moderate Resolution Imaging Spectroradiometer (MODIS) satellite images that expose spatiotemporal cloud cover dynamics of previously undocumented global complexity. We demonstrate that cloud cover varies strongly in its geographic heterogeneity and that the direct, observation-based nature of cloud-derived metrics can improve predictions of habitats, ecosystem, and species distributions with reduced spatial autocorrelation compared to commonly used interpolated climate data. These findings support the fundamental role of remote sensing as an effective lens through which to understand and globally monitor the fine-grain spatial variability of key biodiversity and ecosystem properties.

## Abstract 2

Analysis of new data summarizing 15 years of satellite imagery reveals global cloud cover dynamics in unprecedented detail, enabling strongly improved ecosystem and species distribution predictions compared to traditional interpolated climate data.

## Author Summary

Cloud cover affects many important ecological processes, including reproduction, growth, survival, and behavior. When quantified globally at high spatial resolution, cloud cover dynamics can provide key information for delineating a variety of habitat types and predicting species distributions. In this study, we develop a new near-global, fine-grain (≈1 km) dataset of monthly cloud frequencies from 15 y of twice-daily satellite images. The new data reveal cloud cover dynamics at unprecedented spatial resolution. We show that the direct, observation-based nature of cloud-derived metrics can improve predictions of habitats, ecosystem, and species distributions with reduced spatial autocorrelation compared to commonly used interpolated climate data. These findings support the fundamental role of remote sensing as an effective lens through which to understand and globally monitor the fine-grain spatial variability of key biodiversity and ecosystem properties. Applications of these new data extend beyond ecology to validation of global climate models, economic applications in solar energy, tourism, and resource planning. With climate and land-use changes expected to perturb the geography of these conditions and ecological connections, standardized satellite-based observation of cloud cover may represent a key avenue for monitoring the health of biodiversity and ecosystems into the future.

## Introduction

Advanced spatial assessment and monitoring of biodiversity in today’s rapidly changing world is vital for managing future biological resources and a key element of several 2020 targets of the Convention on Biological Diversity [1,2] and the Intergovernmental Platform on Biodiversity and Ecosystem Services [3]. Growing evidence highlights the importance of fine-grain (≤1 km) climatic and environmental variability in driving the spatial distribution and abundance of organisms [4] and the need to correctly capture this variation globally [5,6]. Ecological research at regional to global extents remains reliant on environmental information that lacks important detail and is often interpolated between ground stations over vast distances of highly variable terrain [5,7].

Cloud cover and precipitation are prime examples of important environmental factors that can have significant spatial variability at grains lower than 2 km [8] and are particularly difficult to interpolate [9]. Cloud cover influences processes ranging from reproductive success in reptiles [10] to leaf wetness [11], CO_2_ uptake [12], and the geographic distribution of plants [13]. Especially in the tropics, seasonal variability of cloud cover is typically more important than day length and solar angle in reducing available solar irradiance, with multi-fold ecological consequences [14]. These effects are difficult to observe in other remotely sensed products including vegetation indices, which for many parts of the world do not show much change throughout the year [15]. For example, [16] reported that persistent cloud cover on Santa Cruz Island (California, United States) reduced annual drought stress in bishop pine (*Pinus muricata*) by 22%–44% compared to less cloudy areas further inland. In other work, [10] experimentally altered available radiation to simulate increased cloud cover and found it lowered maternal pregnancy success and slowed growth rates of female McCann’s skinks (*Oligosoma maccanni*). Furthermore, cloud frequency can be a better predictor than interpolated precipitation for species distributions [13]. However, most studies incorporating cloud observations have very limited spatial extents and required either local cloud observations or extensive processing of satellite observations. Fortunately, several decades of satellite data now offer new opportunities to characterize our planet by providing data globally with consistent methodology and, critically, spatially contiguous observations at high spatial resolution.

To date, the complex processing paths required for extracting fine-grain data from existing cloud products and the coarse spatial resolution of existing climatologies have made it difficult to access and account for cloud dynamics in ecological and biodiversity models. For example, recent work on the ecology of cloud forests [11] required processing over 14,000 daily ungridded satellite images simply to compare cloud frequency at two locations that were 2 km apart. The current alternative to this approach is to rely on available cloud climatologies, which typically have very coarse spatial resolution. A recent systematic review of satellite-derived cloud climatologies [17] and all Moderate Resolution Imaging Spectroradiometer (MODIS) level three atmosphere products are summarized at 1° (≈110 km) resolution (S1 Table). While this grain may be appropriate for study of global cloud dynamics (and necessary for cross-platform comparison), it is far too coarse to capture fine-grain variability that is important in many ecological questions [11]. Furthermore, cloud dynamics are particularly difficult to adequately parameterize in climate models [18] and, thus, the quality of modeled fine-grain cloud products are questionable. There are a few examples of finer-grain climatologies based on other sensors, such as HIRS (≈20 km) [19], AVHRR PATMOS-x [20] (≈11 km), and GridSAT [21] (≈8 km), but these are eight to 20 times coarser than possible with MODIS observations (S1 Table). There have been several regional–national climatologies assembled at finer (≤1 km) resolution from MODIS data, and these generally perform well in comparison with station observations and other meteorological satellites (e.g., [22]).

To date, there have been two efforts to produce large-domain, high-resolution (≤1 km) cloud climatologies from the MODIS archive. One is based on the MOD35 250 m visible cloud mask [23], but this is spatially bounded to the tropics and incorporates only seven years of data (2000–2006). Additionally, these data were derived from the problematic collection 5 MODIS (MOD35) cloud mask and, thus, contain significant land-cover and processing-path biases in cloud frequency [24]. The other MODIS-derived 1 km cloud climatology [25,26] avoids the problematic MOD35 algorithm through a simple cloud masking procedure based on scaled visible wavelength (RGB) images from the MODIS “Rapid Response” system [27]. Douglas et. al. developed an algorithm that applies a user-defined threshold to convert RGB “brightness” to “cloudiness.” However, the product is based on a derivative of surface reflectance data rescaled for visual appeal [27], is strongly dependent on the brightness threshold, and is problematic over high-albedo surfaces (such as urban areas or snow). Furthermore, this approach does not exploit more sophisticated tests used in most cloud detection algorithms such as cloud-top infrared temperature [17] and is only available for select regions around the globe.

In this paper, we develop new fine-grain (≈1 km resolution) global cloud climatologies from the 15-year MODIS archive of twice-daily observations. We then validate the new layers using cloud observations collected at a global network of 5,388 weather stations since 1971. To illustrate the utility of the new global fine-grain cloud cover observations, we explore four biodiversity science applications: (1) Biome boundaries are typically characterized by multiple environmental attributes, but cloud-associated variables tend to be either statistically interpolated or not well captured in existing global datasets. We illustrate how the substantial fine-grain variation in cloud cover dynamics can help delineate sharp ecological transitions. (2) Climatic stability is associated with elevated biological endemism [28–30] but is difficult to map globally at high spatial resolution. We suggest that cloud cover is an important yet often overlooked variable and put forward a first map of cloud cover stability. (3) Some ecosystems and the species specialized on them are particularly affected by local cloud conditions. We demonstrate how the newly developed remote sensing products allow a delineation of cloud forest habitats worldwide in unprecedented detail and with the potential for continued monitoring. (4) Species distribution modeling is an important biogeographical tool, but is often reliant on interpolated climatic data. We show that incorporating remotely sensed cloud climatologies in species distribution models can improve predictive accuracy without inflating autocorrelation inherent in commonly used interpolated data.

## Results and Discussion

**Fig 1:**
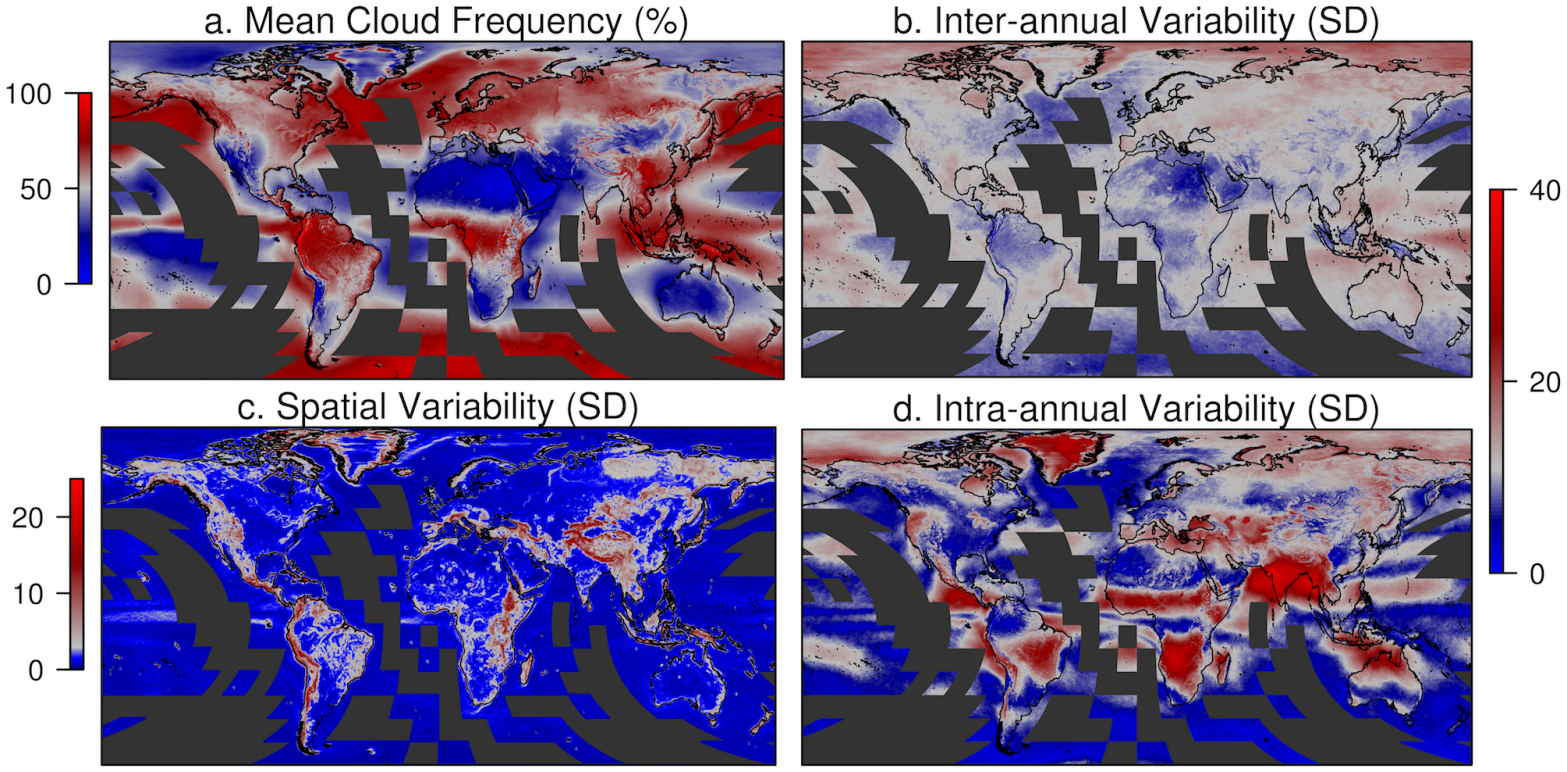
Global 1 km cloud metrics. **A**. Mean annual cloud frequency (%) over 2000–2014. **B** Inter-annual variability in cloud frequency (mean of 12 monthly standard deviations). **C**. Spatial variability (standard deviation of mean annual cloud frequency within a one-degree, ≈110 km, circular moving window). **D** Intra-annual variability in cloud frequency (standard deviation of 12 monthly mean cloud frequencies). Grey indicates the (**A)** median global cloud frequency (51%) and (**B,D**) median inter-annual variability (11%), blues indicate areas with below-median values, and reds indicate areas with higher-than-median values. Data are available only for MODIS land tiles, resulting in missing data in black tiles over oceans. For further exploration see http://earthenv.org/cloud and for download see http://doi.org/10.6084/m9.figshare.1531955.

The new 1 km dataset (see http://www.earthenv.org/cloud for online exploration) confirms equatorial South America, the Congo River basin in Africa, and Southeast Asia as the cloudiest regions of the world, with annual cloud frequencies (proportion of days with a positive cloud flag) ≥80% (Fig 1A). But, in contrast to existing evidence (S1 Table), the new product captures the frequency of cloud cover at substantially increased spatial resolution. In many regions (often but not always mountainous), cloud cover varies starkly over very short distances (Fig 1C), revealing variability hidden in spatially aggregated cloud products currently used in ecosystem, biodiversity, and climate modeling that are >100–10,000 times coarser (S5 Fig and S1 Table) [8,31–33]. See Materials and Methods for more details on validation.

While elevation per se is often associated with orographic cloud formation and used to support interpolation of cloud-related variables such as precipitation (S5B Fig), the global correlation between the new mean annual cloud frequency and elevation is limited (Spearman’s ρ = -0.196, *n* = 2 x 10^8^ for all MODCF; Spearman’s ρ = -0.256, *n* = 1,558 for station data [34]). In turn, a strong statistical relationship between mean monthly cloud frequency and mean monthly precipitation measured at meteorological stations (1950–2014: Spearman’s ρ = 0.74, *n* = 243,186; 2000–2014: ρ = 0.64, *n* = 55,253; GHCN v2) points to the utility of high-resolution remotely sensed cloud information itself for precipitation estimation.

### Temporal Variation and Hotspots of Cloud Stability

Regions also vary strongly in the temporal variability of cloud cover, both within and between years (S3 Table and S6 Fig). Away from the poles, the biomes with the highest mean annual cloud frequency are the tropical and subtropical forests (80.1% ± 9.5 mean and standard deviation of monthly cloud frequencies across the biomes; see [35], Fig 1, and S3 Table). The same biomes also have extremely low intra-annual variability (defined here as the standard deviation of the 12 monthly mean cloud frequencies) of 5.6% ± 3.3 and 5.9% ± 2.4, respectively. Cloud intra-annual variability is highest over the monsoonal biomes of India (29.6% ± 3.1) as well as the Sahel and southern mid-latitudes (Fig 1D). The largest inter-annual variability (defined as the mean of the inter-annual standard deviations for each month) outside Antarctica occurs in the tropical and subtropical savannas and the shrublands of Oceania (14.4% ± 2.2) and Australasia (13.2% ± 1) (Fig 1B). The mountains in these regions also experience some of the world’s highest spatial variability in cloud frequency (Fig 1C).

**Fig 2:**
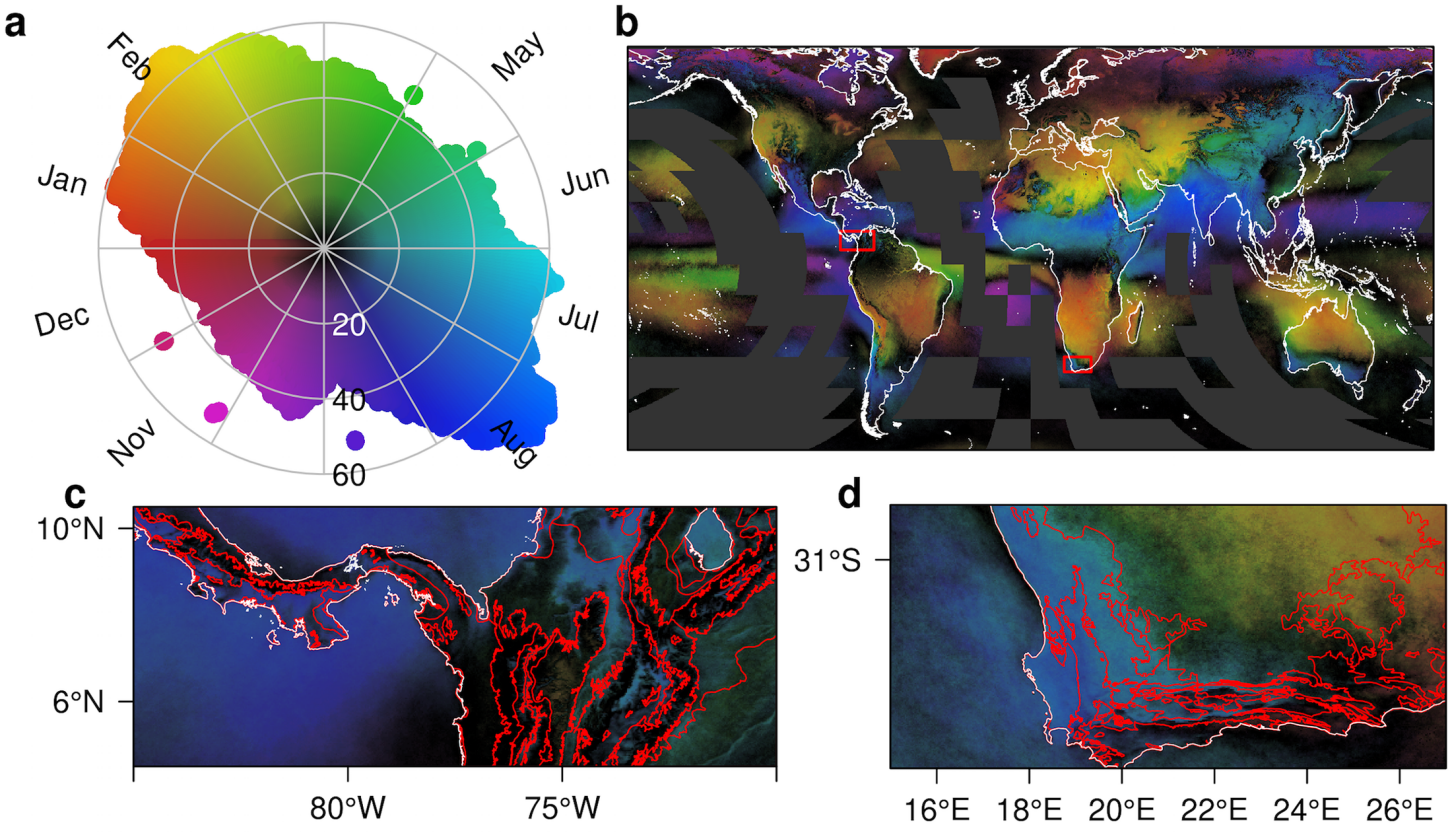
Seasonal cloud concentration. **A.** Color key illustrating the distribution of global cloud seasonality and concentration. The hue indicates the month of peak cloudiness, while the saturation and value indicate the magnitude of the concentration ranging from 0 (black, all months are equally cloudy) to 100 (all clouds are observed in a single month). **B.** Global distribution of seasonal cloud concentration with two red boxes indicating the locations of panels **C** and **D.** Coastlines shown in white, areas with no data are dark grey. **C.** Regional plot of northern South America illustrating the transition from June–July–August to December–January–February cloudiness with little seasonality (dark colors) at high elevations. **D.** Regional plot of southern Africa illustrating the transition from the Mediterranean climate in the southwest to the summer rainfall region in the northeast. Note the incursions of summer clouds and associated rainfall (red colors) along the southern coast. In **C** and **D**, red lines indicate ecoregion boundaries [35]. For further exploration see http://www.earthenv.org/cloud and for download see http://doi.org/10.6084/m9.figshare.1531955.

We quantify the often dramatic fine-scale spatiotemporal dynamics in cloud cover through a metric of cloud “seasonal concentration” [36] that combines the magnitude and timing of seasonal fluctuations in cloud frequency. It illustrates the often remarkably sharp transitions between many of the world’s terrestrial ecosystems (Fig 2). This includes the transition (shown as red lines) along the Isthmus of Panama between Atlantic (black) and Pacific moist forests (blue) and a gradient from peak cloudiness in June–August in the west to December–February in the east (Fig 2C). In another region, the gradient from the winter rainfall Mediterranean climate of southwestern South Africa (blue colors) to the summer rainfall region in the northeast (reds and yellows) is apparent, as well as the band of low seasonality (black) between them (Fig 2D).

**Fig 3:**
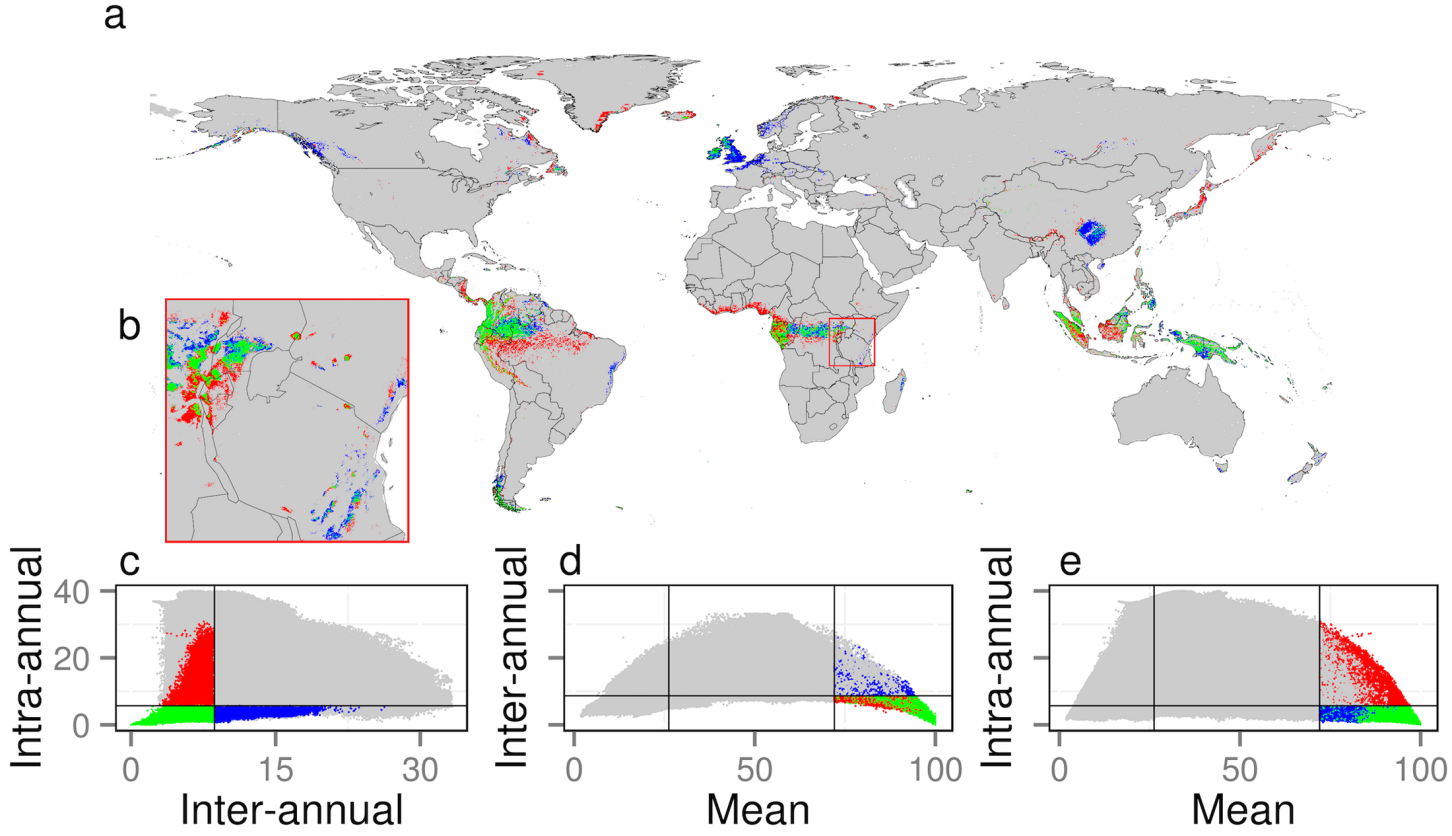
Global hotspots of temporal cloud cover constancy. **A**. Geographic locations of minima in cloud dynamics using colors defined in **C–E**. **B**. Inset showing detail (red square in **A**) over East African Biodiversity Hotspots. **C–E.** Scatterplot of pixels in **A** and **B,** which serves as a color key to the map. Colored pixels indicate locations in the top 10% quantile of mean annual cloud frequency (see **D** and **E**) and bottom 10% quantile of intra-annual cloud variability (blues), inter-annual cloud variability (reds), or both (greens). Lines in scatterplot indicate the 10th (and 90th for mean annual) percentiles. For further exploration see http://www.earthenv.org/cloud and for download see http://doi.org/10.6084/m9.figshare.1531955.

Low temporal variability in an environment characterizes many refugial areas of high endemism, which, in turn, may support regional origination and maintenance of biodiversity [28–30]. While the spatial grain of contiguous endemism or richness data (≈100 km for birds [37]) is currently not sufficient for a global test in 1 km detail, collection of finer-scale data and downscaling modeling techniques [38] hold promise for utilizing this high-resolution intra- and inter-annual cloud variation for identifying and monitoring potential climatic refugia for species survival. Hotspots of within- and among-year cloud stability highlight known centers of endemism in the tropics (e.g., the Andes, Congo River Basin, Indonesia, Borneo, and New Guinea, Fig 3). For example, much of the Eastern Afromontane biodiversity and endemism hotspot of Uganda, Rwanda, Kenya, and Tanzania [28,29] is associated with extremely high cloud climatic stability (Fig 3B), yet is restricted and fragmented in extent. Delineation and monitoring of the detailed occurrence of such locations is vital for understanding how global climate change, in combination with nearby habitat loss, may alter local climate conditions with critical consequences for the continued existence of these specialized local ecosystems.

### Delineating Cloud Forest Distributions

**Fig 4:**
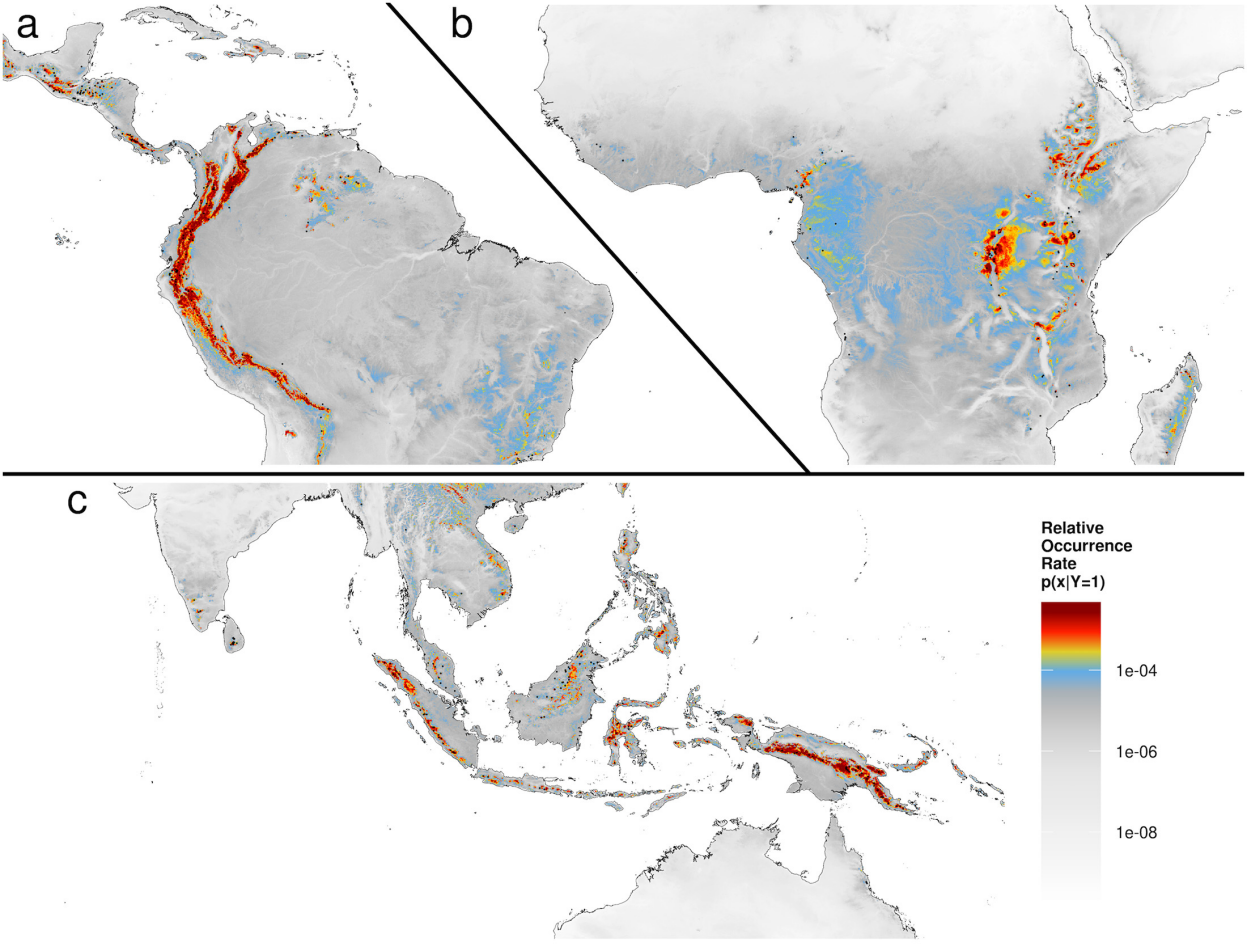
Tropical montane cloud forest distribution. **A–C**: Distribution (relative occurrence rate) of tropical montane cloud forests estimated using an inhomogeneous point process model [47] of 529 cloud forest locations (black points) [48] with the new cloud metrics and elevation [7] (see Materials and Methods and S4 Table for modeling details). Panels show predictions for (**A**) South and Central America, (**B**) Africa, and (**C**) Southeast Asia/Australia. All panels share the color bar shown in panel **C**. For further exploration see http://www.earthenv.org/cloud and for download see http://doi.org/10.6084/m9.figshare.1531955.

One ecosystem particularly strongly influenced by temporal cloud cover dynamics is the cloud forest [11,39,40]. Cloud forests harbor thousands of species specialized on this habitat worldwide and are recognized centers of endemism [41,42]. They are also under increasing threat from human encroachment and climate change [39,43]. Work to date has used remote sensing only over small spatial extents to address this vegetation type [40] or relied on climatic interpolations instead of observations [39]. We tested the utility of the newly developed cloud variables to capture the current distribution of tropical montane cloud forests (Fig 4 and S4 Table). While surely missing important cloud characteristics (e.g., cloud base height [40]), the new cloud cover product provides both a standardized global perspective and the capability of near real-time monitoring of this important habitat. The cloud metrics resulted in an improved model of cloud forest distributions (ΔBIC = 69; point biserial correlation coefficient increase of 0.15) compared to the interpolated variables (mean annual temperature, precipitation, and precipitation seasonality) typically used to delineate this type of ecosystem (S4 Table) [39].

Rapidly changing climate is likely to affect the biophysical conditions required by cloud forest vegetation. Here, we show that remote sensing offers a quantitative capability of monitoring cloud forests worldwide. The models derived here for data from the near-global and continuing MODIS satellite mission, or future refined versions, offer the ability to translate observed changes in mean or intra-annual cloud cover into potential impacts on cloud forest distributions. Such fine-scale cloud cover changes may arise from regional climate change or anthropogenic land cover change. Landcover transformation, including deforestation [32], irrigation [44], and even urbanization [45], can lead to regional hydrological changes, including cloud cover frequency [46]. More work and additional remote sensing products are needed for a rigorous and detailed cloud-forest monitoring system, but the demonstration here shows the potential of bringing remote sensing to this important and urgent task.

### Species Distributions

To date, use of fine-grain, remotely sensed information for large-scale biodiversity applications has been largely limited to vegetation indices and has not included climatological observations, which are typically interpolated from station locations. We illustrate the potentially substantial improvement afforded by the new cloud climatologies by contrasting modeled species distribution predictions from traditional interpolated temperature and precipitation data [7] with those based on the presented satellite-derived cloud information for two species: the montane woodcreeper (*Lepidocolaptes lacrymiger)*, an arboreal songbird of northwestern South America, and the king protea (*Protea cynaroides*), a charismatic shrub found only on moist slopes in the Mediterranean shrublands of South Africa (S7 Fig) [49].

**Fig 5:**
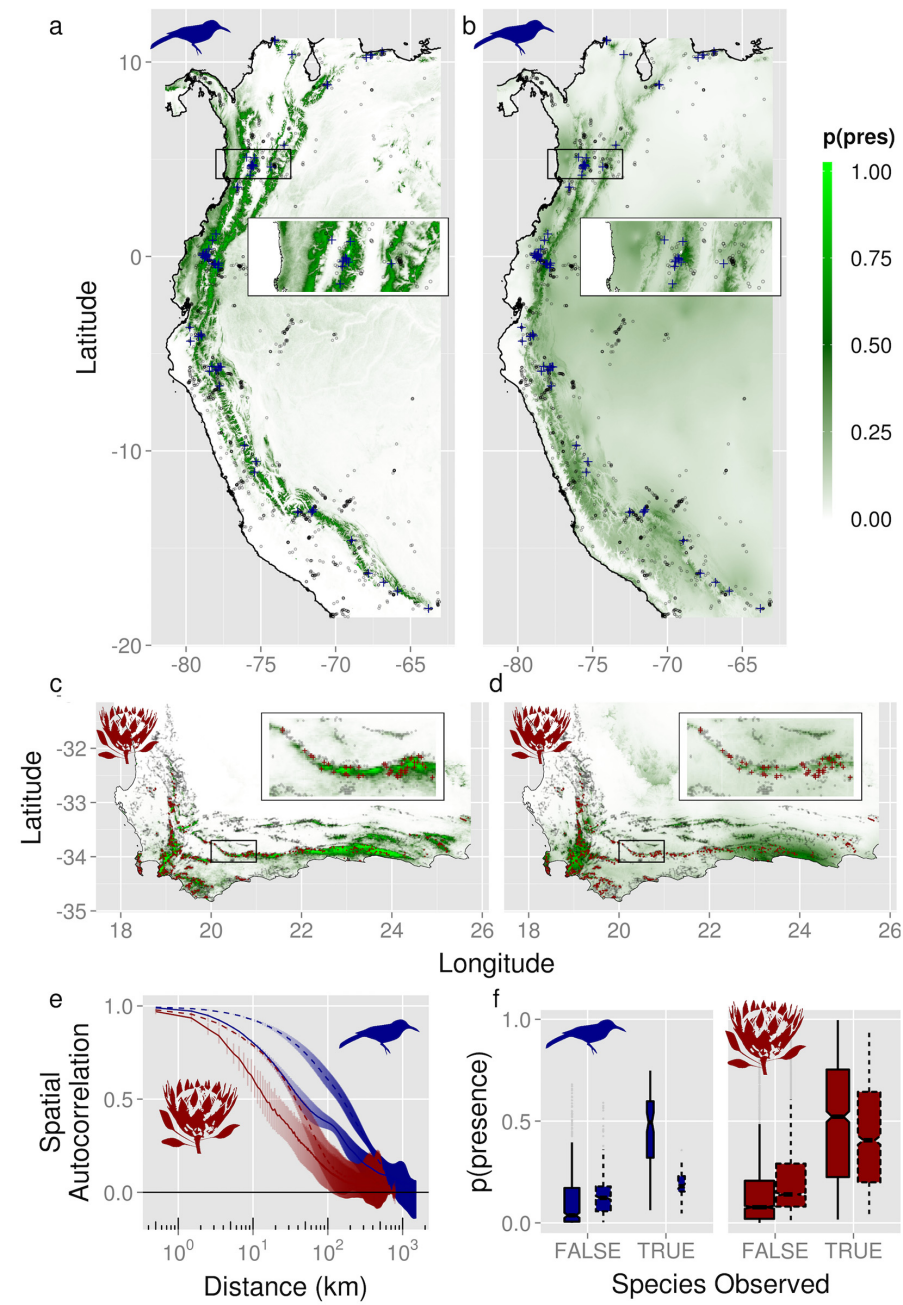
Evaluation of cloud data in species distribution models. **A–F.** Evaluation of predictions from species distribution models of (**A,B,E,F**) montane woodcreeper (*Lepidocolaptes lacrymiger*, blue symbols) and (**C,D,E,F)** king protea (*Protea cynaroides*, red symbols). **A,C** are estimated probability of presence from species distribution models fit using cloud frequency, while **B,D** use interpolated precipitation. Insets in **A–D** show detail from boxed region. Gray points indicate locations with non-detections, while red/blue “+” marks indicate observed presences. **E**. correlograms of the spatial autocorrelation of the data in **A–D,** in which solid lines indicate models built with cloud data (**A,C**) and dashed lines indicate predictions from a model built using interpolated precipitation data (**B,D**). **F**. Estimated probability of presence, in which the species has been undetected at locations with at least five trials or observed (colors/lines as in **E**), box widths proportional to the number of observations. Data available at http://doi.org/10.6084/m9.figshare.1531955.

Remotely sensed cloud information substantially improved model performance for both species for all metrics (for example, the Deviance Information Criterion [DIC] decreased by 315, and the area under the receiver operating characteristic curve [AUC] increased from 0.68 to 0.87 for *L*. *lacrymiger*, and DIC decreased 1,879 and AUC increased 0.82 to 0.87 for *P*. *cynaroides*, Table 1). As predicted, the probabilistic occurrence predictions from models with the cloud data also had much lower and, thus, less inflated spatial autocorrelation than the interpolated precipitation data (Fig 5E, Table 1). Furthermore, the cloud-refined estimates of occurrence probabilities result in estimated range sizes 43% and 18% smaller than those derived from the interpolated precipitation data for *L*. *lacrymiger* and *P*. *cynaroides*, respectively. This illustrates that reliance on coarse and/or interpolated datasets can result in overestimation of the spatial homogeneity of abiotic conditions driving the distribution and likely abundance of organisms. The more substantial model improvement seen for *L*. *lacrymiger* in the Andes is likely due to the less dense weather station network there, as typical for many developing countries, which results in higher uncertainties in the interpolated precipitation layer. As a result, these new cloud layers will likely have the greatest impact in developing countries. The extension of remotely sensed global information beyond vegetation indices to high-resolution climatic conditions represents a major step toward more ecologically relevant, integrative, spatiotemporally contiguous capture and monitoring of biodiversity.

**Table 1:** Evaluation of species distribution models using interpolated precipitation or cloud product for the montane woodcreeper (*Lepidocolaptes lacrymiger*) and king protea (*Protea cynaroides*).

species	model	nPresence	nTrials	AUC	cor	DIC	MPSRF	MoransI	GearyC
*Lepidocolaptes lacrymiger*	Cloud	215	4318	0.87	0.35	1861.15	1.02	0.92	0.08
*Lepidocolaptes lacrymiger*	Precipitation	215	4318	0.68	0.10	2176.33	1.00	0.97	0.03
*Protea cynaroides*	Cloud	3012	18878	0.87	0.52	21258.07	1.01	0.82	0.17
*Protea cynaroides*	Precipitation	3012	18878	0.82	0.40	23137.50	1.00	0.90	0.08

nPresence is the total number of observed presences. nTrials is the total number of trials (species survey/checklists). AUC is the area under the receiver operating curve, a measure of model performance. Cor is the correlation between the observed data and the modeled probability of presence. DIC is the deviance information criterion. MPSRF is Gelman and Rubin's multivariate potential scale reduction factor measuring model convergence. MoransI and GearyC are metrics of spatial autocorrelation. Data available at http://doi.org/10.6084/m9.figshare.1531955.

## Conclusion

Remotely sensed information has the potential to revolutionize our understanding of spatial ecoclimatological patterns and processes through direct capture of environmental variation at fine spatial grain and global extent [50]. Here, we have shown how global cloud dynamics can be quantified in unprecedented spatial detail and that cloud-associated factors are significantly associated with the distribution of various aspects of biodiversity habitats over large spatial scales. In this study, we did not set out to test specific mechanistic connections between vegetation or organisms and clouds, which are reliant on information about other factors (e.g., solar radiation, relative humidity, and soil moisture) not yet attainable at this scale, detail, and reliability. Instead, we focused on tests and demonstrations of the key model-based applications in basic and applied ecology. But we note that the new dataset provides a novel perspective into the fine-grain spatial variability of an environmental factor known to be mechanistically important in several ecological processes [10–13,16,50]. This type of spatially consistent environmental dataset is critical for rigorous hypothesis testing in geographical ecology and biogeography and best-possible prediction and monitoring of habitats and species distributions [51].

Our study also demonstrated key shortcomings of typical interpolated climate surfaces, which can misrepresent biological spatial autocorrelation, leading to false estimates of spatial biodiversity patterns and overestimates of species range size. This illustrates the important role of standardized, long-term, and globally contiguous remote sensing to help close key knowledge gaps and facilitate monitoring of biodiversity [52]. Continued global capture of spatiotemporal cloud cover dynamics has the potential to track the associated often-sharp ecological transitions and the ecological constraints on species ranges they impose, including and beyond those due to precipitation. Applications of this type of information extend beyond ecology to validation of global climate models [18], economic applications in solar energy [53,54], tourism [55], and resource planning. With climate and land-use changes expected to perturb the geography of these conditions and ecological connections, standardized satellite-based observation of cloud cover may represent a key avenue for monitoring the health of biodiversity and ecosystems into the future.

## Materials and Methods

### Calculation of Cloud Frequencies

The MODIS MOD09 atmospherically corrected surface reflectance product includes an internal cloud mask in the PGE11 program that relies on two reflective and one thermal test [56–58]. The reflective tests include the shortwave and middle infrared data combined in the “middle infrared anomaly” index (MIRA = ρ_20,21_ − 0.82ρ_7_ + 0.32ρ_6_, where ρ indicates MODIS band number). The second test uses reflectance at 1.38 microns (1.38 mic = ρ_26_). The MIRA and the 1.38 mic reflectance are designed to be complementary, with MIRA efficiently detecting low or high reflective clouds [56], while 1.38 mic effectively detects high (and potentially not very reflective) clouds. Additionally, a thermal test is used to identify pixels with high infrared reflectance anomalies (e.g., fires, sun-glint, and high albedo surfaces) with respect to near-surface (2 m) air temperature computed by the NCEP reanalysis model [59]. The MOD09 cloud algorithm was designed to minimize confusion over snow and ice by taking the surface air temperature into account. Like many cloud masks, the MOD09 detection algorithm has a binary response (cloudy/not cloudy) and does not retain an estimate of confidence in cloud state (i.e., probability that the pixel was actually cloudy given the tests). The other MODIS cloud mask (MOD35) converts the continuous probabilities into four bins (certainly clear, probably clear, probably cloudy, and confidently cloudy) and is available at the satellite swath level (which would avoid any sampling problems introduced by the orbital parameters and the MODLAND selection criteria). However, due to spatially heterogeneous application of cloud tests (including recently reprocessed Collection 6), the MOD35 mask is unsuitable for generating global and spatially consistent maps of cloud frequencies at 1 km resolution [24]. Liu and Liu introduced an interesting alternative method of estimating cloud cover based on multi-year time series of MOD09 surface reflectance, which is promising but currently based on the frequency of clouds between 8 d MODIS Land Team (MODLAND) composites and, thus, cannot estimate the true daily cloud frequency (e.g., a cloudy observation in a single 8 d MODLAND window indicates eight cloudy days, but a clear observation could indicate one to seven clear days) [60]. Other approaches have been developed to estimate continuous probabilities rather than binned classifications [61], but these have not yet been applied to MODIS data.

We extracted the daily cloud flags from bit 10 of the daily daytime surface reflectance product “state 1 km” Scientific Data Set (SDS) from both the Terra (MOD09GA, collected at approximately 10:30 AM local time) and Aqua (MYD09GA, approximately 1:30 PM) satellites from February 2000 to March 2014 archives (approximately 260 TB of data, n_days_ = 1 x 10^4^, n_pixels_ = 7.2 x 10^8^, n_observations_ = 7.4 x 10^12^). The time series of monthly cloud frequencies (proportion of days with a positive cloud flag) was calculated separately for the daily MOD09GA and MYD09GA data using the Google Earth Engine application programming interface (http://earthengine.google.org/).

### Removal of Albedo and Orbital Artifacts

The MODIS orbit results in systematic gaps in the daily global coverage near the equator [62] that results in nearly longitudinal orbital artifacts (15° for Terra and 345° for Aqua) in the long-term cloud frequencies. To remove these features, we used the Variational Stationary Noise Removal approach (VSNR [63]), a Bayesian image restoration technique. The VSNR is well suited to remove these artifacts because it allows specification of the shape and scale of known artifacts. We used a gabor filter with y = 200, x = 5, and θ = 15 for Terra and θ = -15 for Aqua to minimize the orbital artifacts (S8 Fig).

Inspection of the resulting monthly climatologies revealed anomalously high cloud frequencies over some areas with high maximum and intra-annual variability of albedo, such as the Kati Thanda–Lake Eyre (28.34°S, 137.26°E) in Australia and the Salar de Uyuni salt flats (20.27°S, 67.40°W) of Bolivia. Mean monthly albedo from the 1 km MODIS MCD43B3 product was used to identify and gap-fill in these problematic regions.

### Calculation of Seasonal Metrics

The bias-corrected monthly climatologies for both sensors were then averaged by month and transformed to geographic coordinates at 30-arc-sec spatial resolution (≈1 km). Averaging cloud observations from both products was necessary to minimize scan line artifacts due to gaps in the satellite orbits. Let *m* index months (*m∈*1: 12) and *y* index years (*y∈* 2000: 2014). The combined product represents monthly mean midday cloud frequencies (proportion of days within each month with a positive cloud flag including both Terra and Aqua observations). These monthly time series were summarized to “climatological” mean cloud frequencies and standard deviations: μ_m_ = mean(CF_m,y_) and σ_m_ = SD(CF_m,y_). The inter-annual variability was calculated as mean(σ_m_) and intra-annual variability (seasonality) as SD(μ_m_).

For example, the monthly mean for January, μ_1_, represents the proportion of twice-daily observations (%) with a positive cloud flag out of all observations in January from both Terra and Aqua across all years 2000–2014. The inter-annual standard deviation for January, σ_1_, is the standard deviation of the monthly cloud frequencies observed in each of the 15 Januaries (2000–2014). Due to the algorithm’s use of tests based on reflectance data, the flag is only available for daytime scenes and, thus, high latitudes have no data during winter months (May–September in the Southern Hemisphere and November–February in the Northern Hemisphere).

We also quantified the seasonality of cloud frequencies following [36] by considering mean monthly cloud frequencies as polar vectors with magnitude μ_m_ and direction (month of year expressed in polar coordinates). The sum of the twelve vectors encapsulates both the seasonal concentration (magnitude) and direction (season of maximum cloudiness) for each pixel. Dividing the magnitude by the mean annual cloud frequency results in an index ranging from 0 (equal cloud cover throughout the year) to 100 (all observed clouds occurring in a single month).

### Validation

The monthly CF were validated using a global observational dataset of synoptic weather reports collected at 5,388 stations [34] from 1971 to 2009 (S1 Fig). We extracted the mean “total cloud” amount for each month, which represents the mean proportion of the sky that was covered by all types of cloud during the observations in that month. Comparison of these observations to satellite data must take into account that the sampling radius of these observations (the visible sky) depends on cloud height, cloud thickness, the curvature of the earth, and other factors, but is typically much larger than a single 1 km MODIS pixel. To account for these factors, we took the mean monthly MODCF for a circle with a 16 km radius around each station location, effectively converting the temporal MODCF to mean cloud amount within the sample radius to make it comparable to the station observations [64].

The monthly MODCF (including MODIS data from 2000 to 2014) were compared to station observations using linear models over two time periods: (1) the MODIS era (2000–2009) and (2) the full station record (1970–2009). The MODIS era comparison evaluates the ability of the satellite observations to estimate cloud frequency at station locations during approximately the same time period, while the full station record assesses accuracy and relevance of the 14-year, satellite-derived data for estimating multi-decadal monthly climatologies. For the MODIS era comparison, we included only stations with at least 20 observations per month for the full ten-year period (2000–2009), so the number of stations available was reduced to 1,558 (S2 Table and S1 Fig). For the full record comparison, the station dataset was filtered to include only stations with at least 20 observations per month for at least 20 years, which retained 4,679 stations. Several countries (notably the US, Canada, and New Zealand) converted from human cloud observations to automated laser ceilometers over the past decade, leading to a decline in the number of observations from 1997 to 2009 [34].

The MODCF captures 78% of the variability (*n* = 17,021, RMSE = 7.99% cloud frequency, *p* < 0.001) in monthly mean cloud frequencies observed from the weather stations available with data between 2000 and 2009, with monthly relationships varying from R^2^ = 0.66 (*n* = 1,450, RMSE = 8.30%) in May to R^2^ = 0.84 (*n* = 1,391, RMSE = 7.33%) in November (S2 Fig and S2 Table). The MODCF data is nearly as accurate (R^2^ = 0.74, *n* = 53,678, *p* < 0.001) over the full station record as for the MODIS-era (2000–2009, the period with available validation data) alone.

The spatial distribution of residuals from a linear model between the new MODCF and all station observations (S3 Fig) was used to explore spatial variability of potential biases in the cloud product. While it is tempting to think of the station data as “truth” with which to evaluate the satellite product, the station observations are themselves visual estimates of cloud fraction and prone to regional methodological variability [65,66]. It is also important to note that cloud cover can be significantly affected by land cover [46], which further reinforces the need for high-resolution monitoring of cloud dynamics. The spatial distribution of residuals confirms a known positive bias [67] at high latitudes (>60°), which could be due to the use of modeled surface air temperature in determining the likelihood of snow and ice within the cloud detection algorithm (S3 Fig). There is also a negative bias over the monsoonal regions of India in the Northern Hemisphere summer (JJA, S3 Fig), which is likely due to the diurnal cycle of clouds associated with the monsoon [68] and the timing of MODIS overpasses. We also summarized the residuals over water and different land cover types (S4 Fig). Cloud detection over open water (which tends to be dark and warm in contrast to clouds, which tend to be bright and cold) is relatively straightforward [67], and the role of large water bodies in cloud formation is well understood. In colder months, the cold air above relatively warm water generates instability, increasing cloudiness downwind, while in warmer months the water temperatures are typically cooler than surrounding land, which tends to suppress some cloud types [69].

### Biome Summaries

To illustrate and contrast the spatial variability in cloud frequency within and between Earths biomes, we summarized the mean and standard deviation of the MODCF within each of the up to 14 biomes in each geographic “realm” delineated by the “Terrestrial Ecoregions of the World” dataset [35]. Data are available in S3 Table.

### Cloud Forest Modeling

Locations of known cloud forests were obtained from the Tropical Montane Cloud Forest Sites database maintained by the United Nations Environment Program–—World Conservation Monitoring Centre and published in *A Global Directory of Tropical Montane Cloud Forests* [48]. This dataset contains 529 locations compiled from literature and correspondence with regional experts. Unfortunately, only the central location for each record is known, and there is no accounting for the large variability in size, which can range from 50 hectares to hundreds of square kilometers [48]. We used these locations as presence points with 10,000 randomly selected background points in an infinitely weighted logistic regression (identical to the MaxEnt modeling framework and inhomogeneous point process model [47,70]). We compared models built with elevation, interpolated mean annual temperature, mean annual precipitation, and precipitation seasonality [7] with models built with elevation and the new cloud metrics (including mean annual, inter-, and intra-annual variability) for the global tropical landmass (±23.44° latitude). See S4 Table for more details. Regions were defined with longitudinal breaks at 29.5°W and 63.4°E and included as a factor in the model. Models were compared using AIC, BIC, and the area under the receiver operating characteristic (AUC, S4 Table).

### Distribution Modeling

Occurrence data for *L*. *lacrymiger* were extracted from the eBird Basic Dataset v1.3 [71]. All eBird observations for any species in which the observer recorded looking for “all species” with survey durations of less than 4 h, survey distances of no more than 5 km, and/or a survey area of no more than 500 ha (to reduce spatial uncertainty) were considered as observation “trials,” and observations of *L*. *lacrymiger* were considered “presences.” Occurrence data for *P*. *cynaroides* were extracted from the Protea Atlas Dataset [72]. Survey locations with no observed *P*. *cynaroides* were considered “trials.”

The number of point-level trials and presences for each species were then counted in each 1 km grid cell. To account for imperfect observation (false negatives), the probabilities of occurrence given the various environmental data (see below) were modeled in a Bayesian framework as a zero-inflated binomial using the hSDM.ZIB function in the hSDM R package [73].

We compared two candidate models for each species: the first used spatially interpolated mean annual temperature and three spatially interpolated precipitation measures (mean January precipitation, mean July precipitation, and precipitation seasonality), all from the 30 arc-sec (≈1 km) WorldClim dataset [7]. The second model used the same spatially interpolated mean annual temperature layer with three remotely sensed cloud cover measures (mean January cloud frequency, mean July cloud frequency, and the standard deviation of the intra-annual cloud frequency) (S7 Fig). Models were evaluated by calculating the area under the receiver operator curve (AUC), deviance information criterion (DIC), correlation (cor), and two measures of spatial autocorrelation, Moran’s I (MoransI) and Geary’s C (GearyC). Three chains of each model were burned-in for 10,000 iterations and then run for an additional 50,000 iterations and thinned by 50 to reduce the autocorrelation of the posterior samples. Model convergence was assessed using the multivariate potential scale reduction factor [74]. Range size estimates were calculated by summing the mean posterior pixel-level occurrence probabilities across the modeling domain. This technique avoids the need to apply a threshold the estimated probabilities and accounts for the uncertainty in the model predictions. For example, the estimated range from two cells with 50% probability would be one cell.

## Supporting Information

S1 Fig Validation stations used to validate the cloud climatologies [34]. Black symbols indicate stations with data available only prior to MODIS observations (1970–2000), while red symbols indicate stations with sufficient data from 1970 into the MODIS era (through 2009). North America switched to primarily automatic sensors in the early 2000s, leading to fewer stations with long-term continuous observations. Data available at http://doi.org/10.6084/m9.figshare.1531955.
(TIF)

S2 Fig Mean seasonal (3 mo) cloud amount from 2000 to 2009 from global meteorological stations versus mean 2000–2014 MODCF cloud frequency by season. JJA: June, July, August; SON: September, October, November; DJF: December, January, February; MAM: March, April, May. Least-squares best-fit line (solid), y = x line (dashed), and coefficient of determination are shown in each panel. Colors represent the number of station observations within each grid cell of the scatterplot. Data available at http://doi.org/10.6084/m9.figshare.1531955.
(TIF)

S3 Fig Seasonal residuals from a linear model between station observations and satellite-derived cloud cover. **A–D.** Anomalies at station locations. **E-H.** Anomalies by latitude. **A,E.** DJF: December, January, February; **B,F.** MAM: March, April, May; **C,G.** JJA: June, July, August; **D,H.** SON: September, October, November. Data available at http://doi.org/10.6084/m9.figshare.1531955.
(TIF)

S4 Fig Seasonal residuals from a linear model between station observations and satellite-derived cloud cover divided by land use/land cover (LULC). To account for the spatial scale of the station observations, LULC is the class with the maximum observations within 16 km of the station. Points are jittered residuals under a violin plot [75] illustrating the distribution of residuals in each season. Data available at http://doi.org/10.6084/m9.figshare.1531955.
(TIF)

S5 Fig Comparison of cloud, precipitation, and elevation data from northern South America. **A.** Mean annual cloud frequency (%) for northern South America developed in this paper (≈1 km resolution). **B**. Mean annual precipitation (mm) interpolated from station observations [7]. **C**. Mean annual cloud frequency (%) from PATMOS-x AVHRR data used by the Global Energy and Water cycle Experiment (GEWEX) Cloud Assessment (1 degree, ≈110 km, resolution) [17]. **D**. SRTM Elevation aggregated to 1 km (m). All maps show the region of northern South America illustrated in Fig 2C. Cloud data available at http://doi.org/10.6084/m9.figshare.1531955.
(TIF)

S6 Fig Cloud frequency (proportion of cloudy days within each month) seasonality by geographic realm (columns) and biomes (rows). Colors indicate quantiles: 0%–100% (light grey), 2.5%–97.5% (medium grey), 25%–75% (dark grey), and the median (red). Inset circle indicates area of biome within that realm. Data available at http://doi.org/10.6084/m9.figshare.1531955.
(TIF)

S7 Fig Standardized environmental covariates used in fitting distribution models for *L*. *lacrymiger* (top) and *P*. *cynaroides* (bottom). Data include mean monthly precipitation from January (PPTJAN) and July (PPTJUL), precipitation seasonality (coefficient of variation, PPTSEAS), mean annual temperature (MAT), and SRTM-derived elevation at 1 km resolution (ALT) from WorldClim. Mean monthly cloud frequency in January (CLDJAN), July (CLDJUL), and seasonality (SD of monthly means, CLDSEAS) are from this study. Data available at http://doi.org/10.6084/m9.figshare.1531955.
(TIF)

S8 Fig Comparison of January cloud frequency over the Southwestern Sahara from (A) uncorrected and (B) corrected Terra. Note the banding in the uncorrected data, resulting from variable observation frequency due to orbital artifacts of the MODIS Satellite. Corrected data available at http://doi.org/10.6084/m9.figshare.1531955.
(TIF)

S1 Table Existing satellite-derived cloud-related products with their spatial and temporal grain and extent. To date, there have been two efforts to produce high-resolution (≤1 km) cloud climatologies from the MODIS archive. One is based on the MOD35 250 m visible cloud mask [23] but is spatially bounded to the tropics and incorporates only seven years of data (2000–2006). Additionally, these data were derived from the problematic collection 5 MODIS (MOD35) cloud mask and, thus, contain significant land-cover and processing-path biases in cloud cover frequency [24]. The other MODIS-derived 1 km cloud climatology [25,26] avoids the problematic MOD35 algorithm through a simple cloud masking procedure based on scaled visible wavelength (RGB) images from the MODIS “Rapid Response” system [27]. Douglas et al. developed an algorithm that applies a user-defined threshold to convert RGB “brightness” to “cloudiness.” However, the product is based on a derivative of surface reflectance data rescaled for visual appeal [27], is strongly dependent on the brightness threshold, and is problematic over high-albedo surfaces (such as urban areas or snow). Furthermore, this approach does not exploit more sophisticated tests used in most cloud detection algorithms such as cloud-top infrared temperature [17] and is only available for scattered regions around the globe.
(DOCX)

S2 Table Validation of satellite-derived cloud frequencies using observations from meteorological stations summarized by month and season. Station data were filtered to include only observations during the MODIS era (2000–2009). Month/Season: The temporal aggregation of the data prior to validation. The three-letter acronyms represent three-month seasonal means (DJF: December, January, February; MAM: March, April, May; JJA: June, July, August; SON: September, October, November). Mean: mean cloud frequency across all stations. n: number of station observations used for validation. R^2^: coefficient of determination for a linear model between the satellite-derived and the station cloud frequency climatologies. RMSE: The root-mean-square-errors between the satellite and station data.
(DOCX)

S3 Table Statistics of cloud metrics summarized within each biome as a comma-separated-values (csv) file. Fields are as follows: **icode**: Unique ID for each biome within each geographic realm. **n**: Number of pixels within each biome. **min**: Minimum value of cloud metric within biome. **max**: Maximum value of cloud metric within biome. **mean**: Mean value of cloud metric within biome. **sd**: Standard devation of cloud metric within biome. **product**: Cloud metric (e.g., mean annual, intra-annual, etc.). **meanpsd**: Mean plus standard deviation of value of cloud metric within biome. **meanmsd**: Mean minus standard deviation of value of cloud metric within biome. **code**: Unique code indicating realm and biome. **realm**: Geographic realm from Olson, D. M. et al. Terrestrial Ecoregions of the World: A New Map of Life on Earth. *BioScience* 51, 933–938 (2001). **biome**: Biome name from Olson, D. M. et al. Terrestrial Ecoregions of the World: A New Map of Life on Earth. *BioScience* 51, 933–938 (2001). **area**: Area of biome (km^2^).
(CSV)

S4 Table Comparison of distribution models for tropical montane cloud forests. Regression coefficients and goodness-of-fit metrics for an infinitely weighted logistic regression [47]. MAT: mean annual temperature; MAP: mean annual precipitation; PSeas: precipitation seasonality from [7]. AUC is the area under the receiver operating characteristic curve, COR is point biserial correlation coefficient, AIC is the Akaike information criterion, and BIC is the Bayesian information criterion. Continental regions were defined with longitudinal breaks at 29.5°W and 63.4°E. The region baseline was Africa. Stars indicate coefficient significance: *** *p* < 0.001, ** *p* < 0.01, * *p* < 0.05. Model formulas were as follows. (1) Interpolated Precipitation: cf ~ elev+I(elev^2) +PSeas +MAT+I(MAT^2)+ I(MAP^2) +MAP* region; (2) Cloud Product: cf ~ elev+I(elev^2)+inter+intra+meanannual*region; (3) All: cf ~ MAT+I(MAT^2)+MAP+I(MAP^2)+PSeas+inter+intra+meanannual*region.
(DOCX)

## Abbreviations

AUC: area under the receiver operating characteristic curve
DIC: Deviance Information Criterion
MODIS: Moderate Resolution Imaging Spectroradiometer
