# Supplementary figures and images for: Remotely Sensed High-Resolution Global Cloud Dynamics for Predicting Ecosystem and Biodiversity Distributions
Source: PLoS Biol. 2016 Mar 31;14(3):e1002415. doi: 10.1371/journal.pbio.1002415 (PMC4816575; doi:10.1371/journal.pbio.1002415)

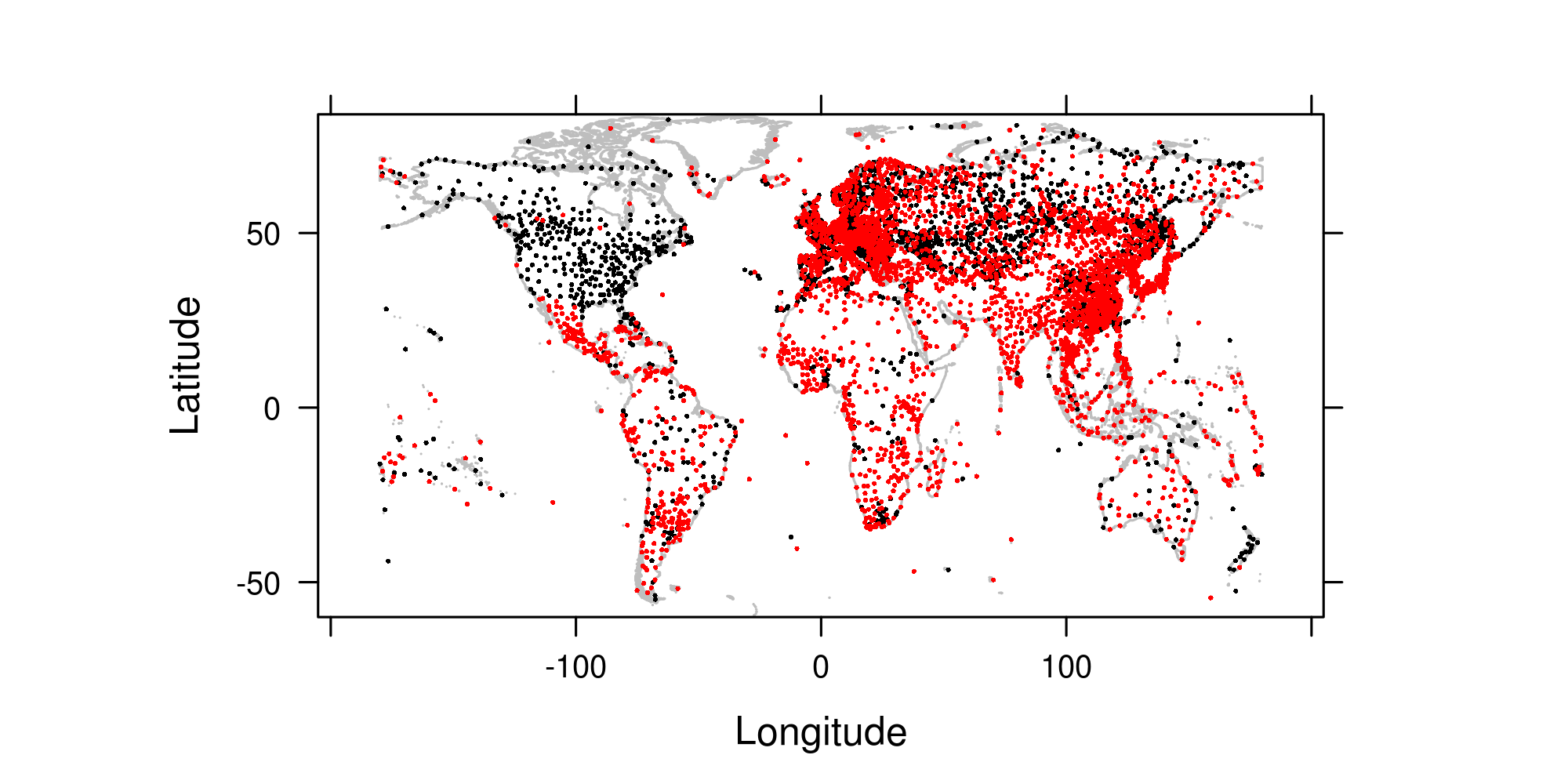

Supplement: S1 Fig — Black symbols indicate stations with data available only prior to MODIS observations (1970–2000), while red symbols indicate stations with sufficient data from 1970 into the MODIS era (through 2009). North America switched to primarily automatic sensors in the early 2000s, leading to fewer stations with long-term continuous observations. Data available at http://doi.org/10.6084/m9.figshare.1531955. (TIF) [file pbio.1002415.s001.tif]

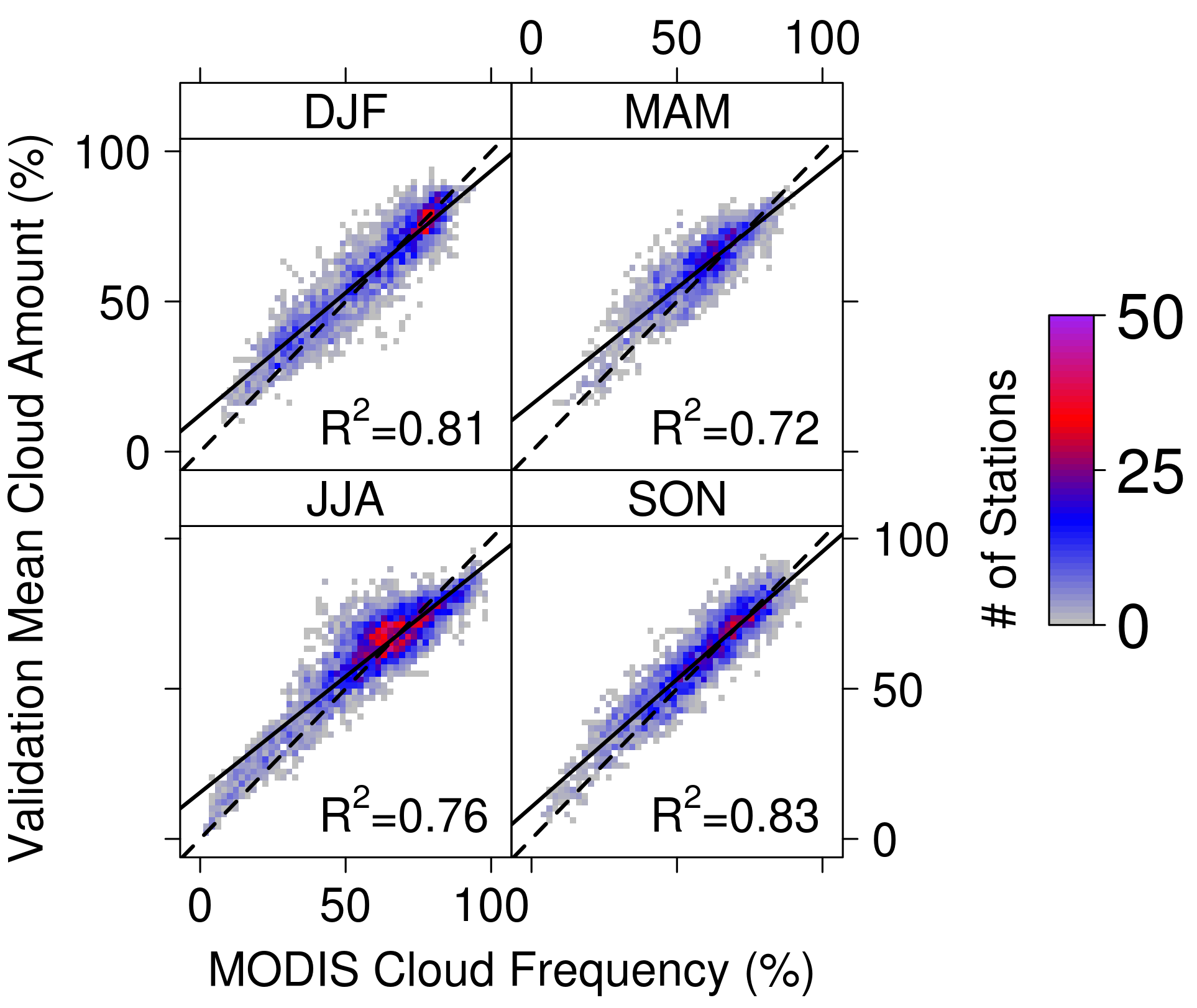

Supplement: S2 Fig — JJA: June, July, August; SON: September, October, November; DJF: December, January, February; MAM: March, April, May. Least-squares best-fit line (solid), y = x line (dashed), and coefficient of determination are shown in each panel. Colors represent the number of station observations within each grid cell of the scatterplot. Data available at http://doi.org/10.6084/m9.figshare.1531955. (TIF) [file pbio.1002415.s002.tif]

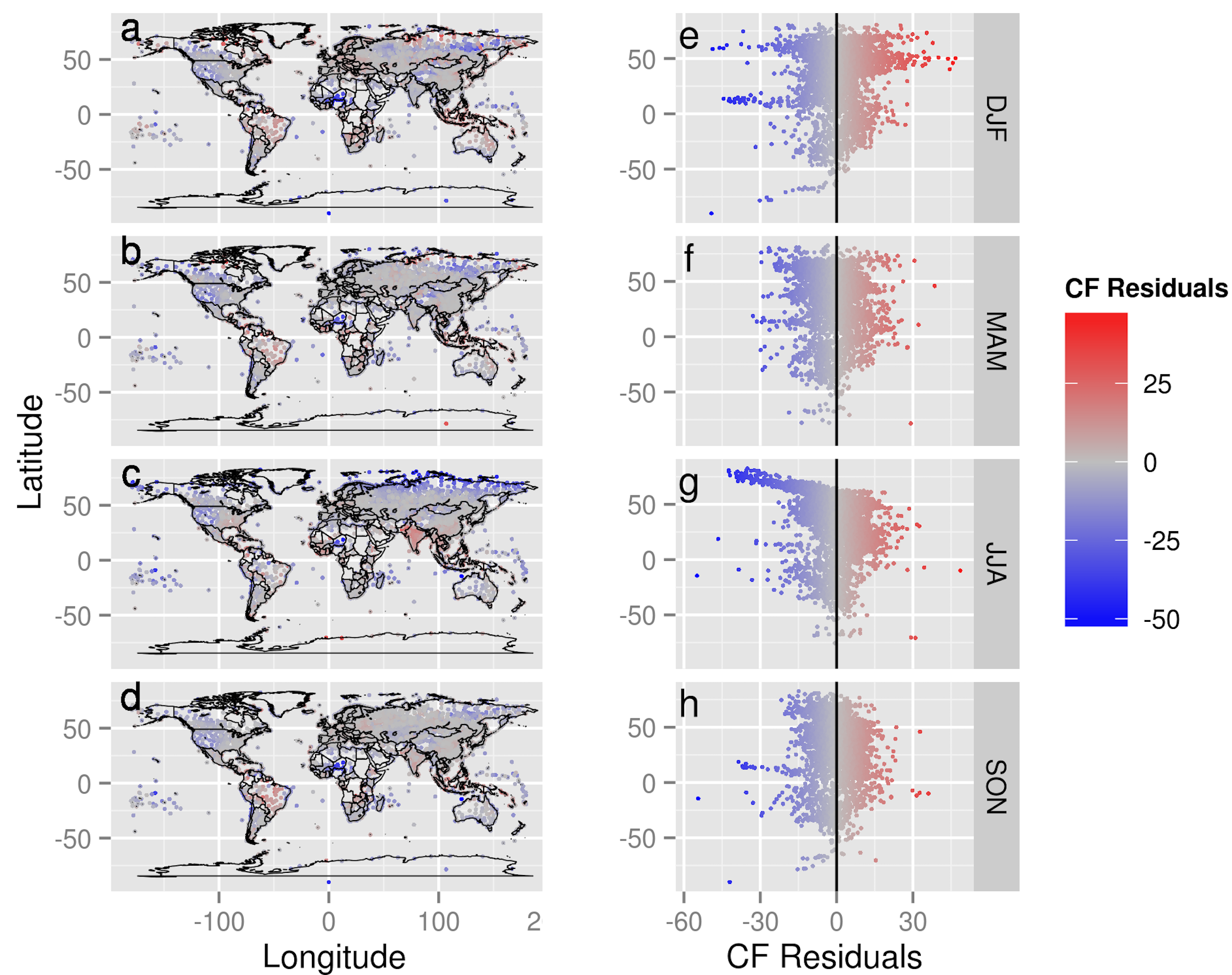

Supplement: S3 Fig — A–D. Anomalies at station locations. E-H. Anomalies by latitude. A,E. DJF: December, January, February; B,F. MAM: March, April, May; C,G. JJA: June, July, August; D,H. SON: September, October, November. Data available at http://doi.org/10.6084/m9.figshare.1531955. (TIF) [file pbio.1002415.s003.tif]

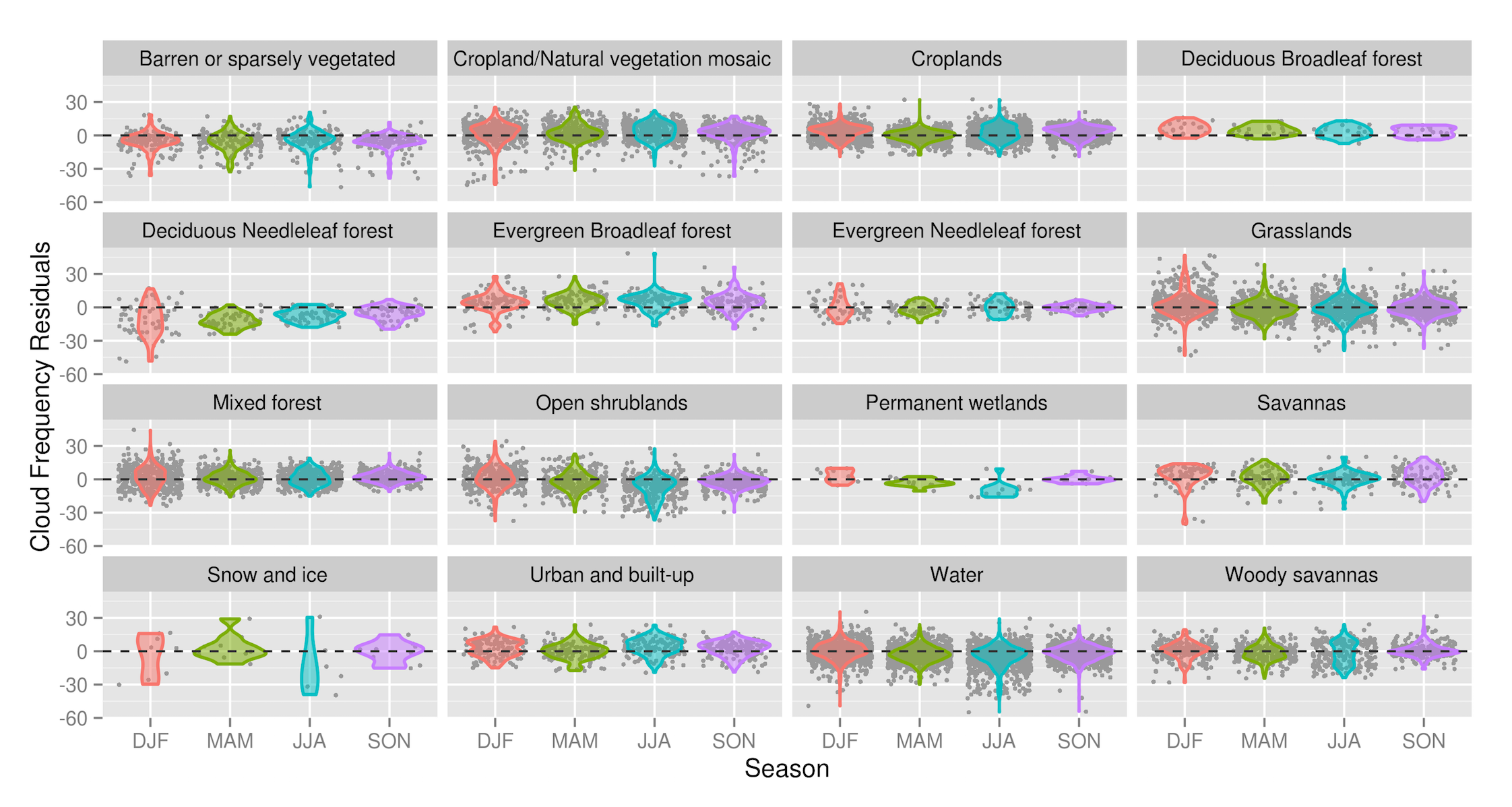

Supplement: S4 Fig — To account for the spatial scale of the station observations, LULC is the class with the maximum observations within 16 km of the station. Points are jittered residuals under a violin plot [75] illustrating the distribution of residuals in each season. Data available at http://doi.org/10.6084/m9.figshare.1531955. (TIF) [file pbio.1002415.s004.tif]

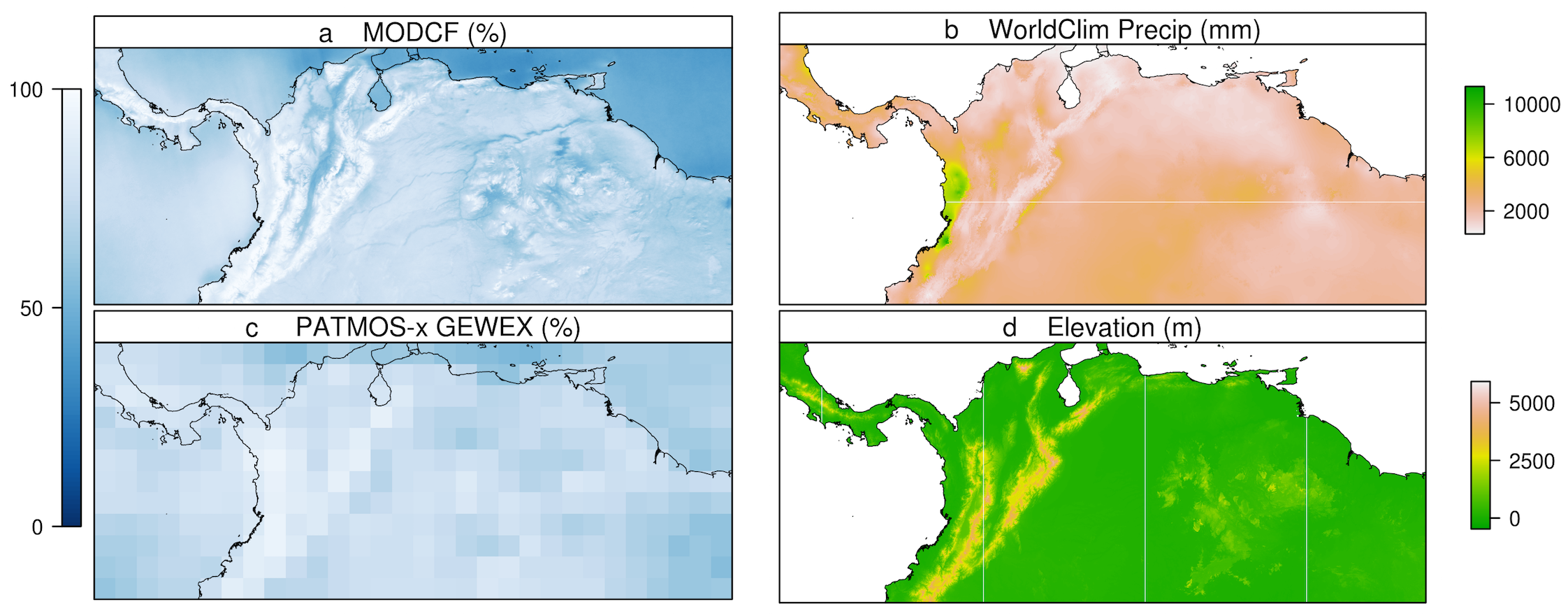

Supplement: S5 Fig — A. Mean annual cloud frequency (%) for northern South America developed in this paper (≈1 km resolution). B. Mean annual precipitation (mm) interpolated from station observations [7]. C. Mean annual cloud frequency (%) from PATMOS-x AVHRR data used by the Global Energy and Water cycle Experiment (GEWEX) Cloud Assessment (1 degree, ≈110 km, resolution) [17]. D. SRTM Elevation aggregated to 1 km (m). All maps show the region of northern South America illustrated in Fig 2C. Cloud data available at http://doi.org/10.6084/m9.figshare.1531955. (TIF) [file pbio.1002415.s005.tif]

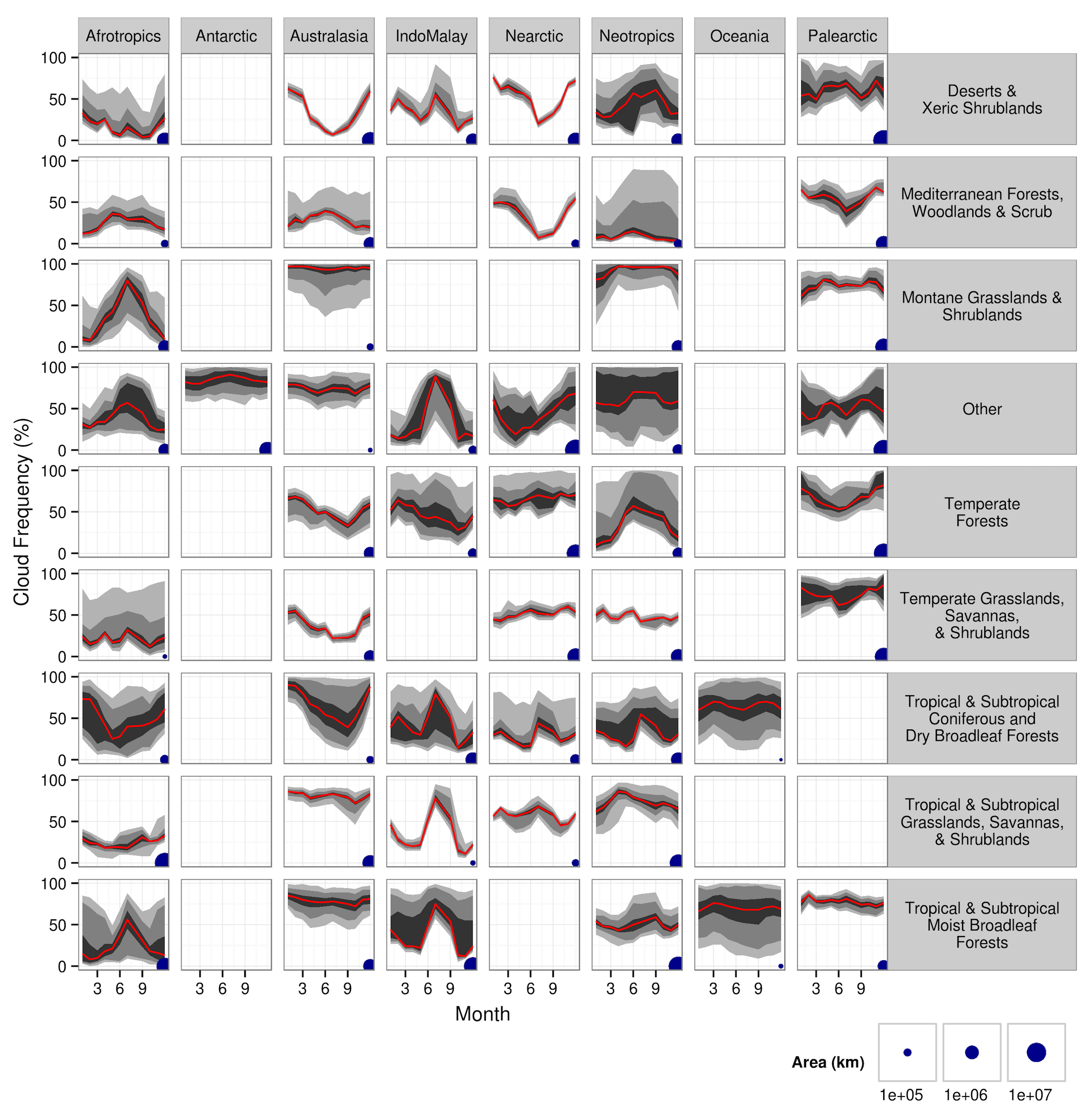

Supplement: S6 Fig — Colors indicate quantiles: 0%–100% (light grey), 2.5%–97.5% (medium grey), 25%–75% (dark grey), and the median (red). Inset circle indicates area of biome within that realm. Data available at http://doi.org/10.6084/m9.figshare.1531955. (TIF) [file pbio.1002415.s006.tif]

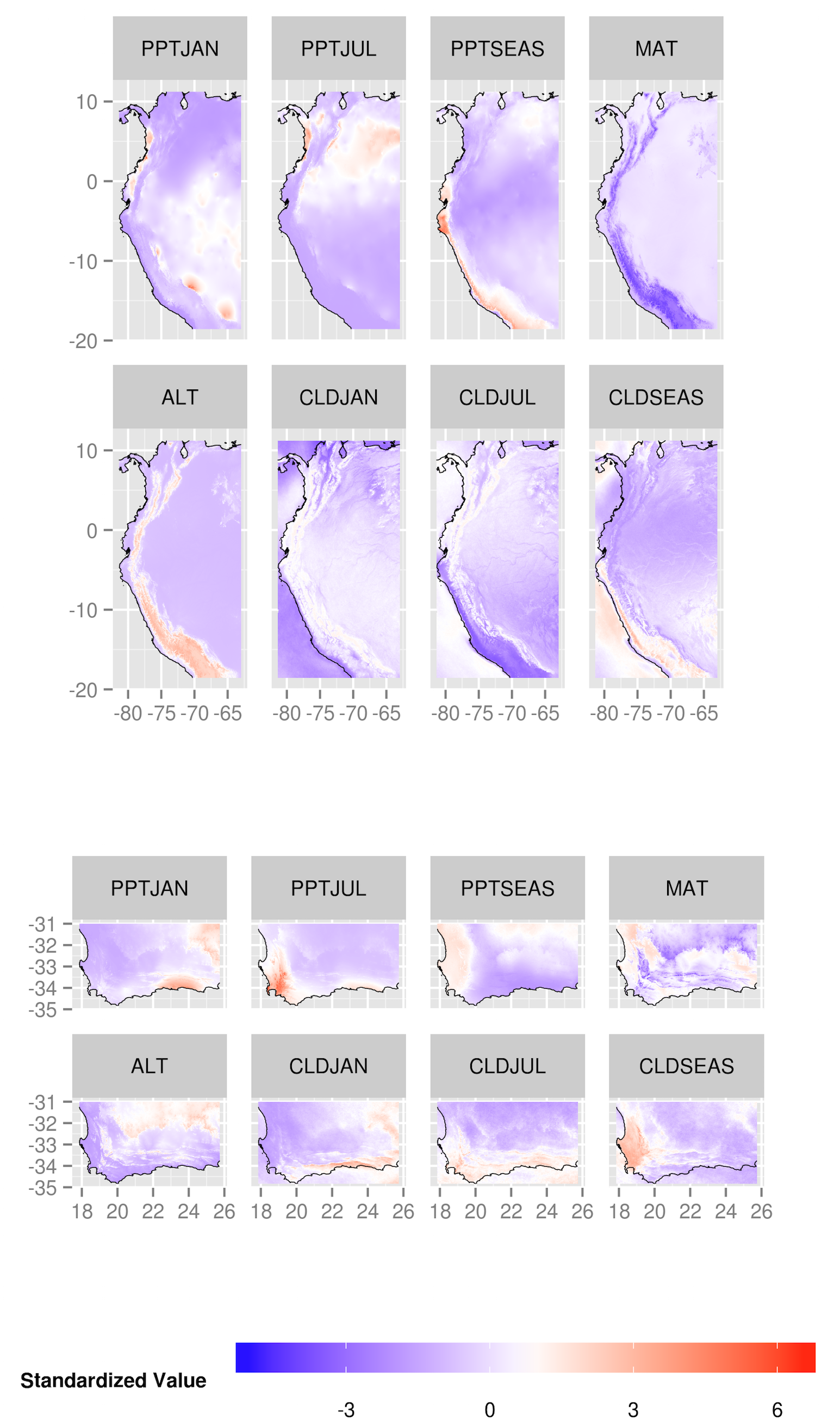

Supplement: S7 Fig — Data include mean monthly precipitation from January (PPTJAN) and July (PPTJUL), precipitation seasonality (coefficient of variation, PPTSEAS), mean annual temperature (MAT), and SRTM-derived elevation at 1 km resolution (ALT) from WorldClim. Mean monthly cloud frequency in January (CLDJAN), July (CLDJUL), and seasonality (SD of monthly means, CLDSEAS) are from this study. Data available at http://doi.org/10.6084/m9.figshare.1531955. (TIF) [file pbio.1002415.s007.tif]

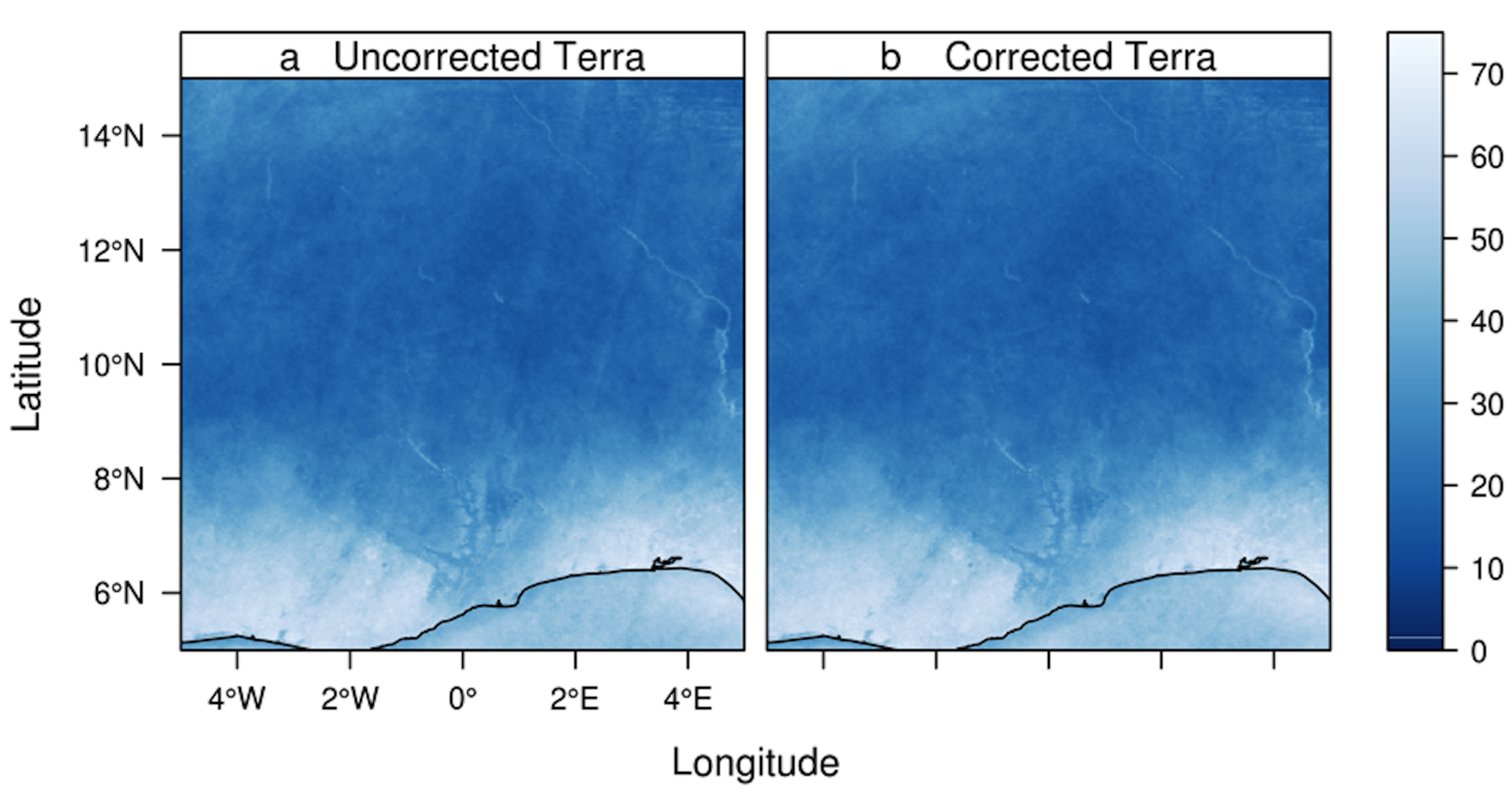

Supplement: S8 Fig — Note the banding in the uncorrected data, resulting from variable observation frequency due to orbital artifacts of the MODIS Satellite. Corrected data available at http://doi.org/10.6084/m9.figshare.1531955. (TIF) [file pbio.1002415.s008.tif]
