# Supplementary material for: Remotely Sensed High-Resolution Global Cloud Dynamics for Predicting Ecosystem and Biodiversity Distributions
Source: PLoS Biol. 2016 Mar 31;14(3):e1002415. doi: 10.1371/journal.pbio.1002415 (PMC4816575; doi:10.1371/journal.pbio.1002415)
Supplement: S1 Table — To date, there have been two efforts to produce high-resolution (≤1 km) cloud climatologies from the MODIS archive. One is based on the MOD35 250 m visible cloud mask [23] but is spatially bounded to the tropics and incorporates only seven years of data (2000–2006). Additionally, these data were derived from the problematic collection 5 MODIS (MOD35) cloud mask and, thus, contain significant land-cover and processing-path biases in cloud cover frequency [24]. The other MODIS-derived 1 km cloud climatology [25,26] avoids the problematic MOD35 algorithm through a simple cloud masking procedure based on scaled visible wavelength (RGB) images from the MODIS “Rapid Response” system [27]. Douglas et al. developed an algorithm that applies a user-defined threshold to convert RGB “brightness” to “cloudiness.” However, the product is based on a derivative of surface reflectance data rescaled for visual appeal [27], is strongly dependent on the brightness threshold, and is problematic over high-albedo surfaces (such as urban areas or snow). Furthermore, this approach does not exploit more sophisticated tests used in most cloud detection algorithms such as cloud-top infrared temperature [17] and is only available for scattered regions around the globe. (DOCX) [file pbio.1002415.s009.docx]

S1 Table: Existing satellite-derived cloud-related products with their spatial and temporal grain and extent.

| Name | Description | Spatial Domain | Spatial Grain | Temporal Domain | Temporal Grain |
| --- | --- | --- | --- | --- | --- |
| GEWEX / ICCP [17] | Compiled from 12 satellite products for comparison study | Global | 1^o^ (≈110km) | 1983-2009 | Monthly |
| HIRS [19] | Cloud frequency from NOAA/HIRS/2 | Global | ≈20km | 1979-2001 | Daily |
| AVHRR PATMOS-x [20] | Cloud product derived from NOAA’s Advanced Very High Resolution Radiometer (AVHRR) | Global | 0.1^o^ (≈11km) | 1981-2010 | Daily |
| GridSat [21] | IR, water vapor and visible bands combined from multiple calibrated geostationary satellites. Not currently available. | 70**°**S - 70**°**N with missing data early in the record | 0.07^o^ (≈8km) | 1980-present | 3-hour |
| MODIS Cloud Climatology [23] | Optical and IR data from MODIS MOD35 | 40**°**S - 40**°**N | 1km | 2000-2006 | monthly, diurnal |
| MODIS Cloud Climatology [26] | Derived from thresholded MODIS RGB images | Scattered regions mostly in tropics | 250m | 2003-present | monthly |

To date, there have been two efforts to produce high-resolution (≤1km) cloud climatologies from the MODIS archive. One is based on the MOD35 250m visible cloud mask [23], but is spatially bounded to the tropics and incorporates only seven years of data (2000-2006). Additionally, these data were derived from the problematic collection 5 MODIS (MOD35) cloud mask and thus contain significant land-cover and processing-path biases in cloud cover frequency [24]. The other MODIS-derived 1-km cloud climatology [25,26] avoids the problematic MOD35 algorithm through a simple cloud masking procedure based on scaled visible wavelength (RGB) images from the MODIS “Rapid Response” system [27]. Douglas, et. al., developed an algorithm that applies a user-defined threshold to convert RGB “brightness” to “cloudiness.” However, the product is based on a derivative of surface reflectance data rescaled for visual appeal [27], is strongly dependent on the brightness threshold, and is problematic over high-albedo surfaces (such as urban areas or snow). Furthermore, this approach does not exploit more sophisticated tests used in most cloud detection algorithms such as cloud-top infrared temperature [17] and is only available for scattered regions around the globe.
