# Supplementary material for: Remotely Sensed High-Resolution Global Cloud Dynamics for Predicting Ecosystem and Biodiversity Distributions
Source: PLoS Biol. 2016 Mar 31;14(3):e1002415. doi: 10.1371/journal.pbio.1002415 (PMC4816575; doi:10.1371/journal.pbio.1002415)
Supplement: S2 Table — Station data were filtered to include only observations during the MODIS era (2000–2009). Month/Season: The temporal aggregation of the data prior to validation. The three-letter acronyms represent three-month seasonal means (DJF: December, January, February; MAM: March, April, May; JJA: June, July, August; SON: September, October, November). Mean: mean cloud frequency across all stations. n: number of station observations used for validation. R2: coefficient of determination for a linear model between the satellite-derived and the station cloud frequency climatologies. RMSE: The root-mean-square-errors between the satellite and station data. (DOCX) [file pbio.1002415.s010.docx]

**S2 Table: Validation of satellite-derived cloud frequencies using observations from meteorological stations summarized by month and season.**

| Month/Season | Mean | n | R^2^ | RMSE |
| --- | --- | --- | --- | --- |
| Annual | 56.60 | 17,021 | 0.78 | 7.99 |
| DJF | 58.60 | 4,185 | 0.81 | 8.10 |
| MAM | 56.36 | 2,879 | 0.72 | 7.59 |
| JJA | 54.85 | 5,726 | 0.76 | 8.54 |
| SON | 57.12 | 4,231 | 0.83 | 7.18 |
| January | 59.01 | 1,403 | 0.80 | 8.40 |
| February | 57.25 | 1,399 | 0.80 | 8.01 |
| March | 56.24 | 1,438 | 0.76 | 7.58 |
| April | 56.47 | 1,441 | 0.67 | 7.57 |
| May | 56.50 | 1,450 | 0.66 | 8.30 |
| June | 55.03 | 1,426 | 0.73 | 8.99 |
| July | 53.76 | 1,428 | 0.79 | 8.83 |
| August | 54.09 | 1,422 | 0.82 | 7.95 |
| September | 55.03 | 1,421 | 0.81 | 7.21 |
| October | 57.00 | 1,419 | 0.83 | 6.83 |
| November | 59.34 | 1,391 | 0.84 | 7.33 |
| December | 59.58 | 1,383 | 0.82 | 7.85 |
|  | | | | |

Station data were filtered to include only observations during the MODIS era (2000-2009). Month/Season: The temporal aggregation of the data prior to validation. The three-letter acronyms represent 3-month seasonal means (DJF: December, January, and February; MAM: March, April, and May; JJA: June, July, and August; SON: September, October, and November). Mean: mean cloud frequency across all stations. n: number of station observations used for validation. R^2^: Coefficient of determination for a linear model between the satellite-derived and the station cloud frequency climatologies. RMSE: The root-mean-square-errors of between the satellite and station data.
