# Supplementary material for: Remotely Sensed High-Resolution Global Cloud Dynamics for Predicting Ecosystem and Biodiversity Distributions
Source: PLoS Biol. 2016 Mar 31;14(3):e1002415. doi: 10.1371/journal.pbio.1002415 (PMC4816575; doi:10.1371/journal.pbio.1002415)
Supplement: S4 Table — Regression coefficients and goodness-of-fit metrics for an infinitely weighted logistic regression [47]. MAT: mean annual temperature; MAP: mean annual precipitation; PSeas: precipitation seasonality from [7]. AUC is the area under the receiver operating characteristic curve, COR is point biserial correlation coefficient, AIC is the Akaike information criterion, and BIC is the Bayesian information criterion. Continental regions were defined with longitudinal breaks at 29.5°W and 63.4°E. The region baseline was Africa. Stars indicate coefficient significance: *** p < 0.001, ** p < 0.01, * p < 0.05. Model formulas were as follows. (1) Interpolated Precipitation: cf ~ elev+I(elev^2) +PSeas +MAT+I(MAT^2)+ I(MAP^2) +MAP* region; (2) Cloud Product: cf ~ elev+I(elev^2)+inter+intra+meanannual*region; (3) All: cf ~ MAT+I(MAT^2)+MAP+I(MAP^2)+PSeas+inter+intra+meanannual*region. (DOCX) [file pbio.1002415.s012.docx]

S4 Table: Comparison of distribution models for Tropical Montane Cloud Forests

| **Group** | **Term** | **Interpolated Climate** | **Cloud Product** |
| --- | --- | --- | --- |
| Intercept and Elevation | (Intercept) | -11.96 (0.15)^***^ | -12.95 (0.24)^***^ |
|  | Elevation | 1.27 (0.23)^***^ | 1.79 (0.08)^***^ |
|  | Elevation^2^ | -0.44 (0.05)^***^ | -0.29 (0.02)^***^ |
| Interpolated Climate | MAT | -1.06 (0.26)^***^ |  |
|  | MAT^2^ | 0.14 (0.06)^*^ |  |
|  | MAP | 2.24 (0.20)^***^ |  |
|  | MAP^2^ | -0.20 (0.04)^***^ |  |
|  | Precipitation Seasonality | -0.28 (0.08)^***^ |  |
| Cloud Product | Inter-annual cloud variability |  | 0.42 (0.07)^***^ |
|  | Intra-annual cloud variability |  | 0.20 (0.10)^*^ |
|  | Mean annual cloud frequency |  | 2.88 (0.24)^***^ |
| Regions | Americas | 0.67 (0.17)^***^ | 1.25 (0.27)^***^ |
|  | Asia Pacific | 0.66 (0.24)^**^ | -1.02 (0.54) |
| Interactions | MAP: Americas | -1.08 (0.21)^***^ |  |
|  | MAP: AsiaPacific | -1.02 (0.23)^***^ |  |
|  | Mean annual cloud: Americas |  | -0.67 (0.22)^**^ |
|  | Mean annual cloud: Asia Pacific |  | 0.95 (0.35)^**^ |
| Goodness of Fit metrics | AUC | 0.94 | 0.95 |
|  | COR | 0.41 | 0.56 |
|  | AIC | 8645.66 | 8591.36 |
|  | BIC | 8732.77 | 8663.95 |
|  | Log Likelihood | -4310.83 | -4285.68 |
|  | Deviance | 8621.66 | 8571.36 |
|  | Num. obs. | 10501 | 10501 |

Regression coefficients and goodness-of-fit metrics for an infinitely weighted logistic regression [47]. MAT: mean annual temperature; MAP: mean annual precipitation; PSeas: precipitation seasonality from [7]. AUC is the area under the receiver operating characteristic curve, COR is point biserial correlation coefficient, AIC is the Akaike information criterion, BIC is the Bayesian information criterion. Continental Regions were defined with longitudinal breaks at 29.5^o^W and 63.4^o^E. The region baseline was Africa. Stars indicate coefficient significance: ^***^p < 0.001, ^**^p < 0.01, ^*^p < 0.05. Model formulas were as follows. 1) Interpolated Precipitation: cf ~ elev+I(elev^2) +PSeas +MAT+I(MAT^2)+ I(MAP^2) +MAP* region; 2) Cloud Product: cf ~ elev+I(elev^2)+inter+intra+meanannual*region; 3) All: cf ~ MAT+I(MAT^2)+MAP+I(MAP^2)+PSeas+inter+intra+meanannual*region.
